# Totally thoracoscopic beating-heart resection of a catheter-related right atrial thrombus: a case report

**DOI:** 10.3389/fcvm.2026.1936906

**Published:** 2026-09-07

**Authors:** Siqi He, Jilu Lang, Chuang Mei, Yuanxiang Wen, Zhifeng Xu

**Affiliations:** 1 Department of Cardiovascular Surgery, Peking University Shenzhen Hospital, Shenzhen, China; 2 Shenzhen University Medical School, Shenzhen, China

**Keywords:** beating-heart surgery, catheter-related right atrial thrombus, multimodality imaging, totally implantable venous access port, totally thoracoscopic surgery

## Abstract

Totally implantable venous access ports (TIVAPs) are widely used to provide reliable vascular access for chemotherapy in patients with malignancies. However, they may rarely give rise to catheter-related right atrial thrombus (CRAT), a potentially life-threatening complication. Here, we report a case of a patient with breast cancer who developed a right atrial thrombus following chemotherapy administered through a TIVAP. As anticoagulation therapy failed to resolve the thrombus, the patient subsequently underwent totally thoracoscopic beating-heart thrombectomy. Multimodality imaging played a pivotal role in localizing the intracardiac mass and characterizing its features, thereby guiding surgical planning. This case demonstrates that totally thoracoscopic beating-heart surgery is a safe and feasible treatment option for CRAT, offering favorable clinical outcomes and excellent cosmetic results, with promising potential for broader clinical application.

## 1 Introduction

Totally implantable venous access ports (TIVAPs) are widely used to provide reliable long-term vascular access for chemotherapy in patients with malignancies, improving both treatment convenience and patient comfort (1). However, TIVAP implantation carries a risk of complications, including infection, thrombosis, and catheter-related dysfunction (1, 2). Among these, catheter-related right atrial thrombus (CRAT) is rare but potentially life-threatening, and no standardized management guidelines have yet been established. Here, we report a case of a patient with breast cancer who underwent TIVAP implantation for chemotherapy and was incidentally found to have a right atrial thrombus during routine follow-up after port removal. As anticoagulation therapy failed to resolve the thrombus, the patient underwent totally thoracoscopic beating-heart thrombectomy, with a favorable postoperative outcome.

## 2 Case presentation

**Figure 1:**
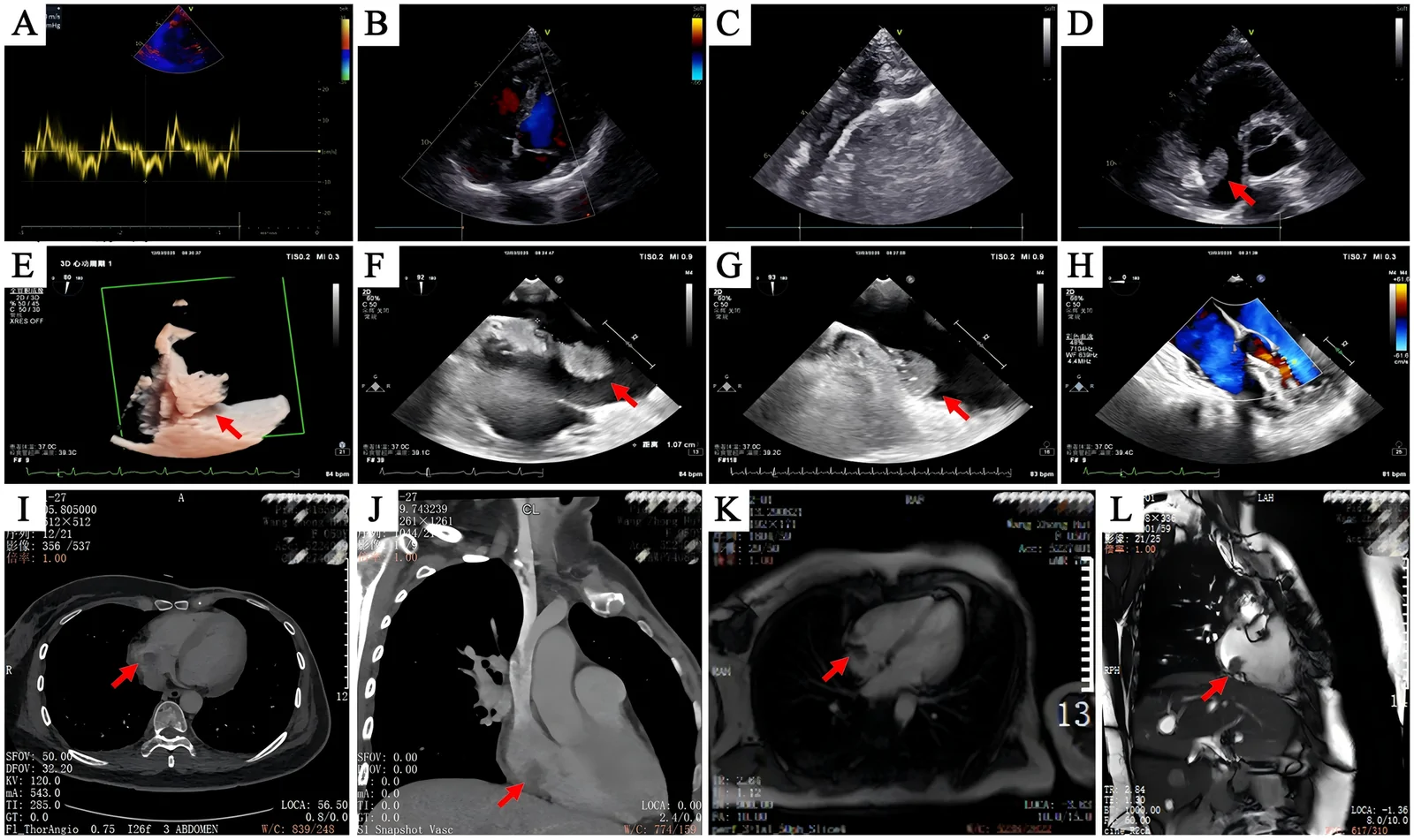
Preoperative multimodality imaging of the right atrial mass. **(A–D)** Transthoracic echocardiography (TTE, Nov.25, 2025) showing a 27 × 16 mm isoechoic mass in the right atrium, extending toward the tricuspid annulus and appearing to originate from the superior vena cava, with an additional 8 mm isoechoic segment within the SVC; left ventricular systolic function was normal (EF 63%). **(E–H)** Transesophageal echocardiography (TEE, Dec.03, 2025) showing an irregular isoechoic mass measuring 21 × 17 mm in the posteroinferior right atrium, with a slightly broad base, well-define margin, heterogeneous internal echogenicity, and scattered punctate hyperechoic foci; the mass exhibited slight mobility during the cardiac cycle and was located approximately 11 mm from the inferior vena cava orifice; a neoplastic lesion could not be excluded. **(I,J)** Cardiac computed tomography venography (CTV, Nov.27, 2025) showing a nodular filling defect in the right atrium (11 × 14 mm), with low-to-slightly-high attenuation on non-contrast images, favoring thrombus. **(K,L)** Cardiac magnetic resonance imaging (CMR, Dec.01, 2025) showing a round, low-signal filling defect in the right atrium (17 × 14 × 20 mm) without enhancement, with slight shape change during the cardiac cycle, suggestive of right atrial thrombus.

A 50-year-old woman was admitted to the Department of Hand, Foot, and Vascular Surgery at our hospital for treatment of varicose veins of the great saphenous vein. During routine preoperative evaluation, transthoracic echocardiography (TTE) incidentally revealed a right atrial mass (Figures 1A–D). The patient was subsequently referred to the Department of Cardiovascular Surgery for further evaluation and management.

**Figure 2:**
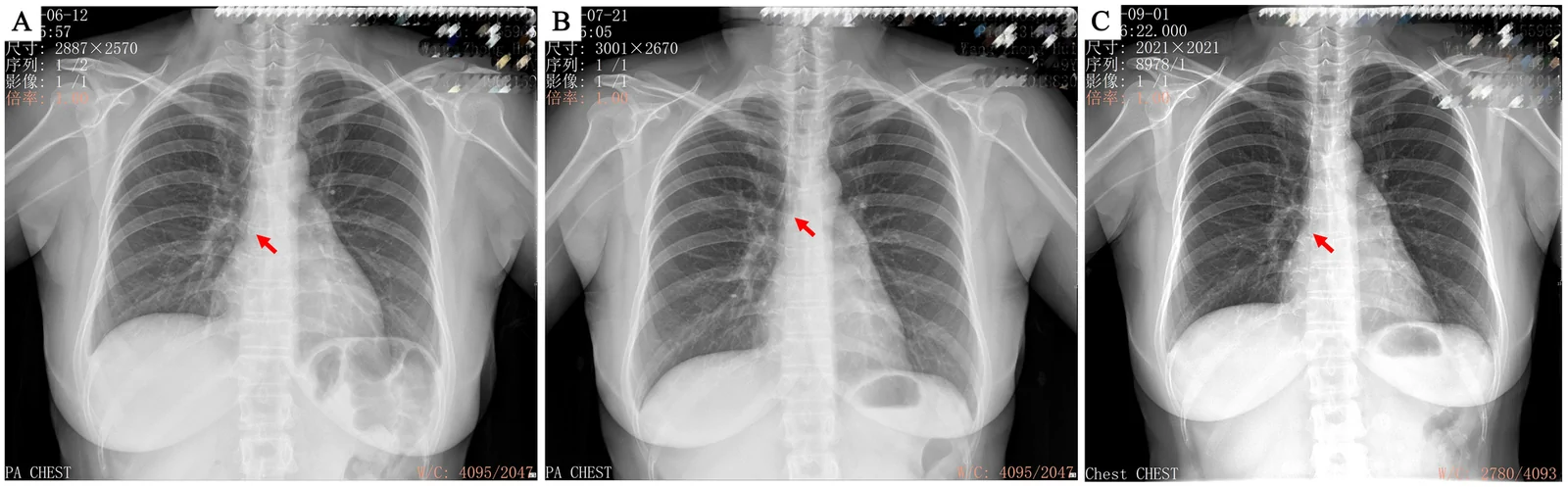
Chest radiographs obtained during TIVAP use, showing sequential changes in catheter tip position: **(A)** immediately after TIVAP implantation, with the catheter tip at the superior border of T8; **(B)** during the third cycle of chemotherapy, with the tip at the T6–T7 level; **(C)** during the fifth cycle of chemotherapy, with the tip at the T8 level.

The patient had a history of breast cancer treated neoadjuvant chemotherapy followed by surgical resection. At the time of admission, she was receiving adjuvant endocrine therapy with anastrozole, abemaciclib, and leuprorelin while awaiting postoperative radiotherapy. For neoadjuvant chemotherapy, a TIVAP had been implanted via the left basilic vein and used to administer six cycles of an ET regimen (epirubicin and nab-paclitaxel) over four months. The patient remained asymptomatic throughout treatment. The catheter tip position was routinely monitored by chest radiography (Figure 2), and no catheter-related dysfunction or other TIVAP-associated complications were observed during its use. The TIVAP remained *in situ* for approximately five months and was electively removed after completion of chemotherapy, three weeks before the current admission.

Laboratory investigations, including coagulation parameters, D-dimer, and tumor markers, were performed (Table 1). Except for an elevated protein C level, all other results were within normal limits. Given the high suspicion of thrombus, anticoagulation with enoxaparin sodium (4,000 anti-Xa IU subcutaneously every 12 h) was initiated as the initial treatment strategy. The patient weighed 55.6 kg and had normal renal function (serum creatinine 62 μmol/L, estimated glomerular filtration rate 94.6 mL/min/1.73 m^2^), corresponding to an enoxaparin dose of approximately 0.72 mg/kg every 12 h. The patient was also advised to maintain strict bed rest to minimize the risk of thrombus embolization.

**Table 1:** Preoperative laboratory findings.

Items	Results	Range
AFP	3.0 ng/mL	0–13.4
CEA	0.8 ng/mL	0–5.0
CA125	8.3U/mL	0.-65.0
CA153	4.4U/mL	0–31.3
CA199	5.2U/mL	0–43.0
SCC	0.6 ng/mL	0–3.2
NSE	8.68 ng/mL	0–25.4
PT	12.20s	11.00–15.00
INR	0.91	0.80–1.20
APTT	31.10s	28.00–43.00
FIB	2.67 g/L	2.00–4.00
TT	16.30s	14.00–21.00
TAT	1.20 ng/mL	<4.00
PAP	0.44ug/mL	<0.80
t-PAI·C	6.80 ng/mL	3.70–9.30
TM	5.30TU/mL	3.80–13.30
AT	80%	80–120
PC	154% ↑	70–130
PS	82%	55–123

To further characterize the right atrial lesion and guide treatment planning, additional multimodality imaging was performed. Duplex ultrasonography of the upper and lower extremities showed no evidence of deep venous thrombosis, and pulmonary computed tomography (CT) angiography revealed no evidence of pulmonary embolism. Contrast-enhanced CT venography demonstrated a filling defect measuring approximately 11 × 14 mm within the right atrium (Figures 1I,J). However, suboptimal contrast opacification of the cardiac chambers limited accurate assessment of the relationship between the lesion and the superior vena cava. Cardiac magnetic resonance imaging (MRI) demonstrated a broad-based lesion measuring approximately 17 × 14 × 20 mm within the right atrium, adjacent to the tricuspid annulus, with slight deformation during the cardiac cycle (Figures 1K,L). After one week of anticoagulation, transesophageal echocardiography (TEE) was performed to further define the lesion's anatomical characteristics and assess its response to treatment. It demonstrated a broad-based right atrial lesion measuring approximately 21 × 17 mm along the posterior-inferior wall of the right atrium, with its inferior margin located approximately 11 mm from the orifice of the inferior vena cava (Figures 1E–H). The differences in lesion measurements across TTE, CTV, MRI, and TEE likely reflected modality-related variability in imaging planes and spatial resolution rather than true interval changes in lesion size.

After one week of therapeutic anticoagulation, the lesion remained essentially unchanged on TEE compared with the initial imaging assessment, suggesting a limited response to short-term anticoagulation. Furthermore, the D-dimer level remained within the normal range throughout (Day 1: 0.38 mg/L FEU; Day 2: 0.35 mg/L FEU; Day 8: 0.29 mg/L FEU). Given the patient's history of breast cancer, the possibility of cardiac metastasis or a primary cardiac tumor could not be entirely excluded. In addition, the lesion remained mobile and was located in close proximity to the tricuspid annulus, raising concern for potential embolization or interference with tricuspid valve inflow, although no hemodynamic obstruction was documented. As the patient wished to proceed with adjuvant radiotherapy without prolonged delay and was unwilling to undergo extended anticoagulation therapy, a multidisciplinary team discussion recommended surgical intervention.

**Figure 3:**
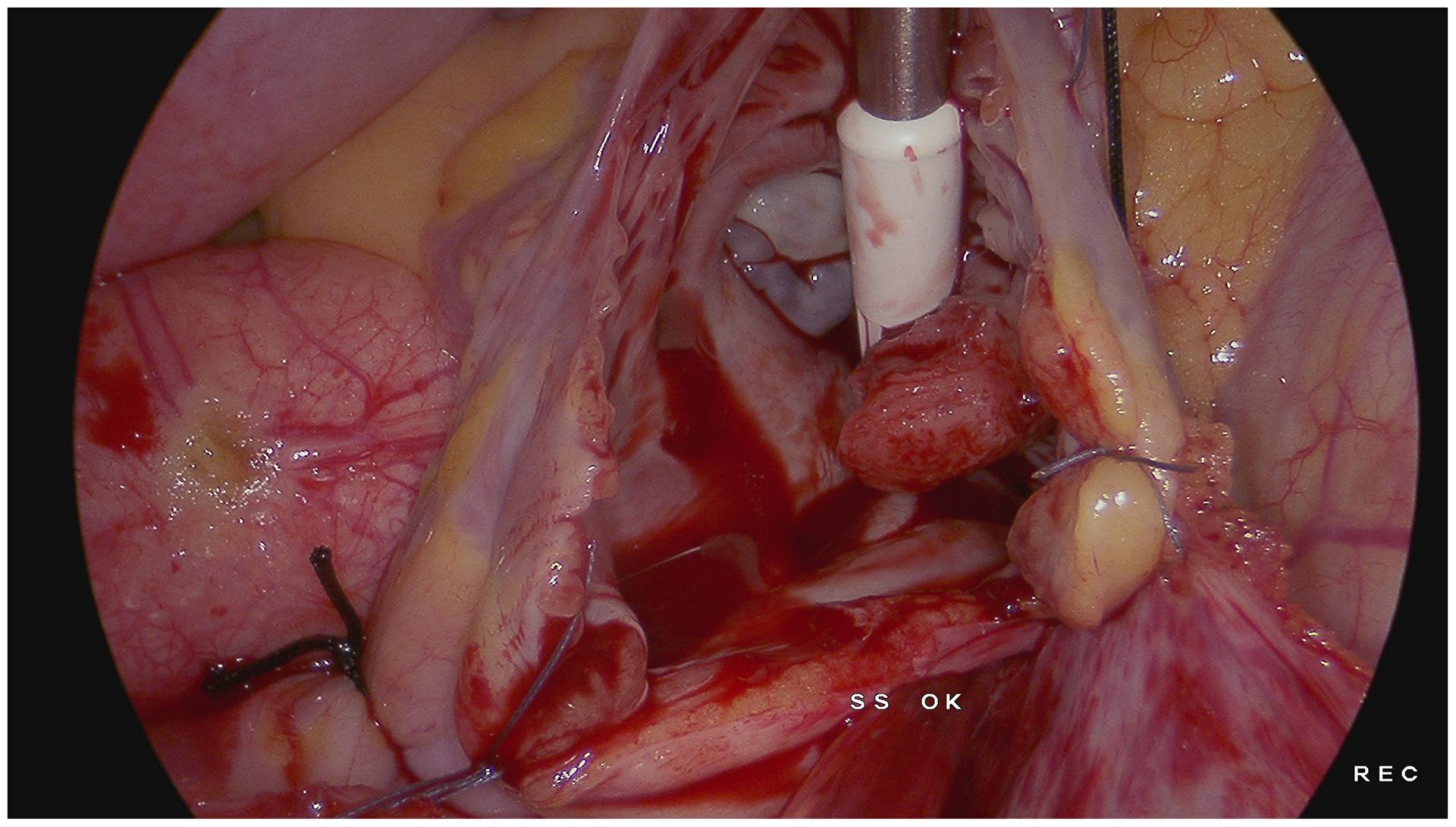
Intraoperative thoracoscopic view of the right atrial thrombus, showing a friable, round mass approximately 25 mm in diameter near the posterior tricuspid annulus, with an irregular surface, a broad base, and firm attachment to the right atrial wall adjacent to the tricuspid orifice.

The patient subsequently underwent totally thoracoscopic beating-heart thrombectomy under cardiopulmonary bypass (CPB). CPB was established via cannulation of the right femoral artery, right femoral vein, and right internal jugular vein and served as circulatory support throughout the beating-heart procedure. Venous return was controlled by temporary occlusion of the superior and inferior vena cavae, and continuous carbon dioxide insufflation was used throughout to reduce the risk of air embolism. Total operative time was 4 h, with a CPB time of 90 min. A thoracoscopic port was created in the fourth intercostal space along the right anterior axillary line, and the main working port was placed in the fourth intercostal space at the right midclavicular line through a submammary incision. After the right atrium was opened under beating-heart conditions, a fragile, irregularly surfaced, broad-based, round mass measuring approximately 2.5 cm in diameter was identified adjacent to the posterior portion of the tricuspid annulus, firmly attached to the posterior-inferior right atrial wall (Figure 3). As the nature of the lesion could not be definitively determined intraoperatively, it was excised *en bloc* together with an approximately 3 × 3 cm portion of the adjacent right atrial wall to ensure complete resection. The resulting right atrial defect was assessed intraoperatively and deemed suitable for primary closure without patch reconstruction. Inspection of the tricuspid valve demonstrated good leaflet coaptation, and no valve repair was required. Estimated blood loss was approximately 100 mL. Autologous blood salvage and reinfusion (469 mL) was performed, and no allogeneic blood products were transfused. Histopathological examination revealed loose fibrinous exudative stroma containing scattered inflammatory cells with focal calcification, consistent with an organized thrombus.

The postoperative course was uneventful, and the patient was discharged on postoperative day 13. Rivaroxaban 10 mg once daily was prescribed postoperative, with a planned duration of 12 months. At the 3.5-month follow-up, TTE showed a mildly dilated main pulmonary artery(29 mm), mild mitral regurgitation, and preserved ventricular systolic function, with no evidence of right atrial thrombus recurrence or tricuspid valve abnormality. D-dimer remained within the normal range. The patient reported good adherence to anticoagulation therapy, with no thromboembolic recurrence or major bleeding events, and continued endocrine therapy throughout the perioperative period. Adjuvant radiotherapy was initiated one month after surgery. A CARE-compliant timeline summarizing the key clinical events, from TIVAP implantation to the final follow-up (Table 2).

**Table 2:** CARE-compliant timeline.

Date	Timeline (Day)	Clinical Event
12 Jun 2025	–	TIVAP implantation via the left basilic vein
12 Jun – 22 Sep 2025	–	Neoadjuvant chemotherapy with six cycles of the ET regimen
14 Oct 2025	–	Breast cancer surgery
7 Nov 2025	–	TIVAP removal
25 Nov 2025	Day 1	Transthoracic echocardiography detected a right atrial mass
27 Nov 2025	Day 3	Cardiac CT venography performed
1 Dec 2025	Day 7	Cardiac magnetic resonance imaging performed
3 Dec 2025	Day 9	Repeat transesophageal echocardiography showed a persistent right atrial lesion
25 Nov–10 Dec 2025	Day 1–16	Anticoagulation with enoxaparin sodium
11 Dec 2025	Day 17	Totally thoracoscopic beating-heart thrombectomy under cardiopulmonary bypass
15 Dec 2025	Day 21	Rivaroxaban 10 mg once daily initiated
24 Dec 2025	Day 30	Discharged from hospital
Jan 2026	Postoperative 1 month	Adjuvant radiotherapy initiated
30 Mar 2026	Postoperative 3.5 months	Follow-up TTE showed no right atrial thrombus recurrence or tricuspid valve abnormality

## 3 Discussion

Right atrial masses are uncommon and encompass both neoplastic and non-neoplastic lesions, with a differential diagnosis that includes thrombus, myxoma, lymphoma, sarcoma, lipoma, and metastatic tumors (3). Because clinical manifestations are often nonspecific, a definitive diagnosis usually requires integration of multimodality imaging findings with histopathological examination. CRAT represents a distinct form of catheter-related thrombosis and, although uncommon, may result in life-threatening complications, including pulmonary embolism, superior vena cava syndrome, and tricuspid valve obstruction (2). With the increasing use of central venous access devices, particularly TIVAPs, the incidence of CRAT has risen. However, because many affected patients remain asymptomatic, its true incidence is likely underestimated (2).

The development of CRAT can largely be explained by Virchow's triad. Repeated mechanical irritation of the right atrial endocardium by the catheter tip may cause endothelial injury, triggering coagulation cascade and platelet aggregation (4–6). In addition, excessive advancement of the catheter into the right atrium may induce local flow disturbance and blood stasis, thereby promoting thrombus formation (7, 8). Furthermore, malignancy itself is associated with a hypercoagulable state, and certain chemotherapeutic agents and endocrine therapy may further increase thrombotic risk (4, 9–11). In the present case, the diagnosis was supported by the temporal association between prior TIVAP placement and removal, the absence of a right atrial lesion before chemotherapy, serial radiographic evidence of catheter tip position, and the anatomical location of the thrombus adjacent to the region potential catheter tip contact.

D-dimer, a degradation product of cross-linked fibrin, is widely used as a biomarker for venous thromboembolism owing to its high sensitivity but relatively low specificity (12, 13). Its diagnostic value, however, may be limited in CRAT, particularly for organized or chronic thrombi. As thrombi undergo organization and fibrosis, fibrinolytic activity declines, resulting in normal or only mildly elevated D-dimer levels. A normal D-dimer result does not exclude the presence of CRAT. In the present case, the D-dimer level remained within the normal range despite the presence of a right atrial mass that was ultimately confirmed by histopathology to be an organized thrombus.

Laboratory investigations and tumor markers have limited value in differentiating right atrial masses. Therefore, multimodality imaging plays a central role in characterizing the lesion, defining its anatomical relationships, and guiding therapeutic decision-making. TTE remains the first-line imaging modality for detecting intracardiac masses because it is noninvasive, readily available, and easily repeatable. In patients with a history of central venous catheterization, particular attention should be paid to the catheter tip and right atrium to improve the detection of CRAT (8). When CRAT is suspected on TTE, TEE provides superior visualization of thrombus morphology, mobility, attachment site, and size, thereby facilitating treatment planning (3, 8). Cardiac CT and MRI offer complementary information when echocardiographic findings are inconclusive. MRI, particularly when combined with multiparametric and contrast-enhanced sequences, is valuable for differentiating thrombi from cardiac tumors (5, 14, 15). In addition, CT with three-dimensional reconstruction provides detailed anatomical information regarding the relationship between the lesion, the venae cavae, and adjacent cardiac structures, which is particularly useful for operative planning. In the present case, sequential TTE, CTV, MRI, and TEE established the diagnosis of CRAT with a high degree of confidence, clarified its relationship with the tricuspid annulus and the venae cavae, and excluded peripheral venous thrombosis and pulmonary embolism. These findings were essential for planning the totally thoracoscopic beating-heart procedure.

Currently, no standardized guideline exists for the management of CRAT. Available treatment options include catheter removal or replacement, anticoagulation, thrombolysis, percutaneous thrombectomy, and surgical thrombectomy (7, 16, 17). For patients with an indwelling catheter, the optimal timing of catheter removal remains controversial. Although removal may improve local hemodynamics and facilitate thrombus resolution, it may also increase the risk of thrombus embolization. Several studies therefore recommend therapeutic anticoagulation before catheter removal whenever feasible (5, 8). Anticoagulation is generally considered first-line treatment for asymptomatic patients with small, non-mobile thrombi (18). Serial echocardiographic follow-up is recommended, and anticoagulation is typically continued for at least six months or until complete thrombus resolution (6, 19). In patients with a poor response to anticoagulation or a large thrombus burden, thrombolysis or percutaneous thrombectomy may be considered in selected cases, although both approaches have important limitations (6, 20).

Percutaneous mechanical thrombectomy or suction embolectomy has recently emerged as a minimally invasive option for selected patients with right atrial thrombi, particularly those at high surgical risk (21–23). However, careful patient selection remains essential. Current evidence suggests that catheter-based aspiration is most suitable for relatively fresh, mobile thrombi, whereas complete removal may be more challenging in patients with large, chronic or organized thrombi, or thrombi with broad-based attachment to the atrial wall (23). In addition, percutaneous approaches primarily provide therapeutic removal but may not yield sufficient tissue for definitive pathological diagnosis. In the present case, the lesion had a broad-based attachment adjacent to the tricuspid annulus, showed no significant response to anticoagulation, and remained of uncertain nature given the patient's recent history of breast cancer. Surgical excision was therefore considered more appropriate, as it allowed complete removal of the lesion together with definitive histopathological confirmation.

Although no consensus guidelines currently define the optimal indications for surgery in patients with CRAT, surgical thrombectomy is generally recommended for large thrombi (typically > 2 cm), failure of anticoagulation therapy, high embolic risk, thrombus infection, superior vena cava obstruction, infective endocarditis, or cases in which an underlying cardiac neoplasm cannot be excluded (5, 24). Surgical intervention may also be reasonable when prolonged anticoagulation is undesirable because of the need for timely treatment of another serious medical condition. The role of PET/CT in differentiating cardiac thrombus from malignancy remains uncertain. Although it may provide additional metabolic information, increased 18F-FDG uptake within the atrial wall or intracardiac lesions is not specific for malignancy. Previous studies have suggested that increased atrial FDG uptake may relate to atrial or ventricular wall hypertrophy, increased pressure or volume overload with upregulation of glucose transporter activity, and myocardial ischemia associated with hypertrophy or hemodynamic stress (25, 26). In addition, organized thrombi may likewise demonstrate variable FDG uptake secondary to inflammatory changes (25, 26).

To date, most reported cases of surgically treated CRAT have been managed through conventional median sternotomy under CPB (27, 28). Although this approach provides excellent surgical exposure, it is associated with greater surgical trauma and a longer postoperative recovery. For our patient, minimizing surgical trauma and facilitating early recovery were particularly important to avoid delaying subsequent oncologic treatment. With advances in minimally invasive cardiac surgery, totally thoracoscopic procedures have become a safe and effective alternative for selected intracardiac diseases. Compared with conventional sternotomy, this approach is associated with reduced postoperative pain, shorter hospital stays, improved cosmetic outcomes, and earlier functional recovery, while maintaining comparable surgical safety (29, 30). The beating-heart technique further avoids aortic cross-clamping and myocardial ischemia-reperfusion injury, thereby maintaining near-physiological myocardial perfusion throughout the procedure and potentially improving postoperative cardiac recovery (31). Totally thoracoscopic beating-heart thrombectomy, however, requires advanced minimally invasive cardiac surgical expertise (30). Continuous cardiac motion increases the technical difficulty of intracardiac manipulation, while maintaining adequate visualization and controlling intracardiac blood return remain challenging. Careful preoperative imaging assessment and appropriate patient selection are therefore essential. This approach may be considered in hemodynamically stable patients with localized right atrial thrombi accessible through thoracoscopic exposure, particularly when anticoagulation fails or prolonged anticoagulation is undesirable. Age alone should not be regarded as a contraindication. Rather, overall physiological status, comorbidities, and anatomical characteristics of the thrombus should guide treatment decisions. Conversely, patients with hemodynamic instability, massive pulmonary embolism, extensive thrombus involving the vena cava or pulmonary artery, or lesions requiring complex intracardiac reconstruction may not be suitable candidates for this minimally invasive approach. In our patient, comprehensive preoperative multimodality imaging clearly delineated the location and attachment of the thrombus, its relationship to the tricuspid valve and venae cavae, and excluded caval thrombosis, thereby providing the anatomical basis for femoral and right internal jugular cannulation and for the totally thoracoscopic beating-heart approach itself. To our knowledge, no previously reported case of CRAT has been treated using this technique. In the present case, the approach proved technically feasible and allowed complete resection of the right atrial thrombus in a carefully selected patient. Histopathological examination confirmed an organized thrombus, and the patient recovered uneventfully, was discharged on postoperative day 13, and resumed adjuvant therapy for breast cancer without delay. This approach may therefore be considered a minimally invasive surgical option in selected patients with CRAT when individualized multidisciplinary assessment supports surgical intervention, although further experience and comparative studies are needed to determine its safety, efficacy, and broader applicability.

In the present case, a right atrial mass was incidentally detected during routine preoperative evaluation in a patient who had undergone TIVAP implantation for breast cancer chemotherapy, with the catheter removed three weeks before admission. Despite the absence of clinical symptoms and persistently normal D-dimer levels, multimodality imaging, including TTE, CT, cardiac MRI, and TEE, progressively characterized the lesion's location, morphology, and mobility. After two weeks of therapeutic anticoagulation, no significant reduction in lesion size was observed. Considering the patient's history of malignancy, the inability to completely exclude a cardiac tumor on imaging alone, and the need for timely initiation of adjuvant radiotherapy, surgical treatment was recommended following multidisciplinary discussion. A totally thoracoscopic beating-heart approach was successfully performed, and histopathological examination confirmed an organized thrombus. Compared with conventional median sternotomy, this minimally invasive strategy provided excellent surgical exposure while minimizing surgical trauma, facilitating rapid postoperative recovery, and allowing timely continuation of oncologic treatment. We believe that, in carefully selected patients with persistent or high-risk CRAT following comprehensive preoperative evaluation, totally thoracoscopic beating-heart thrombectomy represents a safe and feasible treatment option.

This report has several limitations. First, as a single case report, it cannot establish the comparative safety or efficacy of totally thoracoscopic beating-heart thrombectomy relative to conventional surgical or percutaneous approaches. Second, the patient was carefully selected on the basis of the lesion's anatomical characteristics, the absence of peripheral venous thrombosis, and the feasibility of peripheral CPB cannulation. Therefore, these findings may not be generalizable to patients with different thrombus characteristics or more complex cardiovascular conditions. Third, although multimodality imaging and the clinical context supported a thrombotic etiology, the catheter-related origin of the thrombus could not be definitively established. Finally, the follow-up duration was limited, and longer-term follow-up and additional clinical experience are needed to determine the durability and broader applicability of this approach.

## 4 Conclusion

CRAT is a rare but potentially life-threatening complication of central venous access devices, and its diagnosis is often challenging given its typically nonspecific or absent clinical presentation. Multimodality imaging is essential for accurate diagnosis, differentiation from cardiac tumors, and operative planning. Surgical thrombectomy should be considered in patients with thrombi that persist despite anticoagulation or in those at high risk of embolic complications. In appropriately selected patients, totally thoracoscopic beating-heart thrombectomy offers a safe and effective minimally invasive alternative to conventional sternotomy, facilitating rapid postoperative recovery and timely continuation of oncologic therapy.

## Data Availability

The original contributions presented in the study are included in the article/Supplementary Material, further inquiries can be directed to the corresponding authors.
